# C*aryocar brasiliense* supercritical CO_2_ extract possesses antimicrobial and antioxidant properties useful for personal care products

**DOI:** 10.1186/1472-6882-14-73

**Published:** 2014-02-24

**Authors:** Lilian FB Amaral, Patricia Moriel, Mary Ann Foglio, Priscila G Mazzola

**Affiliations:** 1 Department of Clinical Pathology, Faculty of Medical Sciences, University of Campinas (FCM-UNICAMP)–Rua Tessália Vieira de Camargo, 126 Campinas, SP, Brasil; 2 CPQBA–Multidisciplinary Center of Chemical, Biological and Agricultural Researches, University of Campinas (UNICAMP)–Rua Alexandre Cazellato, 999, Vila Betel, Paulínia, SP, Brasil; 3 Rua Horácio Cenci, 345, Apto 32, Campolim, Postal Code: 18047-800 Sorocaba, São Paulo, Brazil

**Keywords:** Pequi, Supercritical CO_2_ extraction, Antimicrobial activity, Antioxidant activity

## Abstract

**Background:**

The cosmetic and pharmaceutical industries have an increasing interest in replacing synthetic antimicrobials in dermatological products due to increased microbial resistance to conventional antimicrobial agents. Pequi *(Caryocar brasiliense)* is a native fruit tree of the Brazilian Cerrado, specifically used in cosmetics, in the food industry, and for medicinal purposes. Leishmanicidal and antifungal activities have been reported previously. This study was designed to evaluate the antimicrobial and antioxidant activities of a *C. brasiliense* extract obtained by supercritical CO_2_ extraction.

**Methods:**

The minimum inhibitory concentrations (MICs) against *Escherichia coli, Pseudomonas aeruginosa*, and *Staphylococcus aureus* were determined by the classical microdilution method. Antiseptic activity against these organisms was evaluated by the plate diffusion method. The antioxidant potential of the extract was evaluated using a method based on the oxidation of 2,2′-azino-bis(3-ethylbenzothiazoline-6-sulphonic acid) (ABTS). The extract’s chemical profile was analyzed for the presence of alkaloids, saponins, anthraquinones, steroids, tannins, flavonoids, and phenolic compounds according to standard colorimetric methods.

**Results:**

The *C. brasiliense* supercritical CO_2_ extract exhibits antimicrobial activity against all bacteria tested. It also possesses antioxidant activity, when compared to a vitamin E standard.

**Conclusions:**

The *C. brasiliense* supercritical CO_2_ extract may be useful for the development of personal care products, primarily for antiseptic skin products that inactivate, reduce, prevent, or arrest the growth of microorganisms with the inherent intent to mitigate or prevent disease as well as products that minimize damage caused by free radicals.

## Background

The cosmetic and pharmaceutical industries have an increasing interest in replacing synthetic antimicrobials in dermatological products due to increased microbial resistance to conventional antimicrobial agents [1,2] in addition to the growing consumer interest in using less aggressive products derived from natural sources. Therefore, there has been renewed research into established plant products of known value that are available to the personal care products industries.

Pequi *(Caryocar brasiliense* Camb) is a typical Brazilian Cerrado fruit tree [3,4]. Its fruit is employed as a vitamin source for culinary purposes and as a source of oil for the manufacture of cosmetics [5]. The word “pequi”, which originates from the Tupi–Guarani language, means “spiny-skinned fruit”, a reference to the shell covered with thin woody spikes, protecting the seeds [6].

Pequi oil is popularly employed for the treatment of hoarseness, sore throat, bronchitis, and cough or as a tonic. It is topically used for dressing wounds, relieving muscle aches, rheumatic pains, and contusions [7]; lung infections and veterinary indications [8]; respiratory problems and scarring [9]; anti-inflammatory activity [10]; as an aphrodisiac, and for the stimulation of bile production [11].

Pequi oil has been reported to contain vitamin A and fatty acids such as palmitic, oleic, myristic, palmitoleic, stearic, linoleic, and linolenic acids [12], which are essential for skin hydration and barrier maintenance, as well as the hydrolipidic mantle [13].

Furthermore, the oil contains linoleic acid, which is used for its bactericidal action in the treatment and/or prevention of open wounds. Linoleic acid is also used to enhance cell membrane permeability, facilitating the entry of growth factors and promoting mitosis and cell proliferation; as well as to stimulate neoangiogenesis and facilitate leukocyte chemotaxis [14].

*In vitro* assays with a hydroethanolic extract from *C. brasiliense* leaves showed antibacterial activity against *Escherichia coli*, *Enterococcus faecalis*, *Pseudomonas aeruginosa*, and *Staphylococcus aureus*. This extract was also shown to possess leishmanicidal and antimicrobial activities, inhibiting the proliferation of *Leishmania amazonensis* promastigote forms*.* This extract also has antioxidant potential analogous to the activities of both vitamin C and rutin [15].

Pequi also contains terpenoids, particularly, carotenoids and polyphenols [16,17]. These metabolites confer skin protection by preventing lipid peroxidation, thereby avoiding free radicals formation, and consequently slowing the cutaneous aging process.

*C. brasiliense* has a high content of phenolic compounds (equivalent to 209 grams of gallic acid per kg). Extracts demonstrate excellent antioxidant activity, scavenging free radicals such as 2,2-diphenyl-1-picrylhydrazyl with an IC_50_ of 9.44 μg/mL and 17.98 μg/mL for aqueous and ethanolic extracts, respectively. The characterization of bioactive compounds responsible for this activity has revealed the presence of potent antioxidants such as gallic acid, quinic acid, quercetin, and *quercetin-3-O-arabinose*[18].

There is a current worldwide interest in finding new and safe antioxidants from natural sources to replace the use of synthetic antioxidants, which can be harmful to human health [19]. Pequi is a fruit found in regions where the trees receive a high incidence of sunlight, which favors the generation of free radicals; moreover, both the pulp and kernel are rich in lipids. These conditions contribute to the biosynthesis of secondary compounds with antioxidant properties, such as phenolic compounds and terpenoids.

Among the antioxidant compounds found in pequi, carotenoids are present in considerable amounts in the pulp, and phenolic compounds, as in other plants and are mentioned as the main substances responsible for the observed antioxidant activity [18].

The majority of the solvents employed in production processes are organic chemicals with hazardous and toxic properties. These solvents are costly, are part of the large waste by-product of the chemical industry, and cause environmental problems [20]. Furthermore, in order to obtain pure products further separation is required. In many cases the regeneration step is the most costly step of the entire process [21].

Mild extraction methods are recommended to preserve the antioxidant and antimicrobial properties of plant extracts; thus, suitable solvents are required to avoid inactivation of phytochemical compounds.

Supercritical CO_2_ extraction has been proposed as a non-polluting method for extracting plant products. In addition to its low toxicity and environmental impact, supercritical CO_2_ extraction replaces conventional extraction methods using organic solvents that require numerous purification processes to remove chemical contaminants. Purity is the biggest advantage supercritical CO_2_ has over all other solvents used for plant extraction [22].

Given the substantial potential of this Brazilian species for wide application in the clinical and cosmetic areas, this study was designed to evaluate the antimicrobial and antioxidant activities of a *C. brasiliense* (Pequi) extract obtained by supercritical CO_2_ extraction.

## Methods

### Plant material

In January 2011, approximately 25 kg of *C. brasiliense* leaves were collected from Montes Claros, Minas Gerais, Brazil. The leaves were dried in an air-circulating oven at 40°C, then ground in a knife mill, and stored in plastic bags at room temperature to protect them from humidity.

Complete leaf samples, representative of the species, were identified by the Herbarium of the University of Campinas (Unicamp), where voucher was deposited under the reference number UEC 150024.

An apolar extract from *C. brasiliense* was prepared by Chemyunion Chemical Ltd., using a supercritical CO_2_ extraction system that consisted of a heated extraction column, CO_2_ and cosolvent pumps, a thermostatic bath, and a pressure gauge.

The activities were conducted with the approval of the Brazilian Institute of Environment and Renewable Natural Resources, which granted access to genetic resources under number 008/2009 (case n. 02001.003785/2011-59).

### Screening of main chemical classes

The phytochemical profile of the crude plant extract was screened using a thin layer chromatography system that tested specific fractions generated, based on varying polarity, during the extraction procedure. This procedure fractionated the crude extract into fiber, a neutral extract, a moderately polar extract, a basic extract, and a polar extract according to the method described by Harborne [23].

The extract’s chemical profile was analyzed for the presence of alkaloids, saponins, anthraquinones, steroids, tannins, flavonoids, and phenolic compounds according to standard colorimetric methods. Compounds from different chemical families were detected by precipitation reactions or staining using reagents specific to each family of compounds.

### Antimicrobial activity

#### Use of microorganisms

The microorganisms tested were established by official microbiology guidelines [24]. Freeze-dried strains were acquired from the Culture Collection Section of the Oswaldo Cruz Foundation. The strains included *S. aureus* ATCC 10390 (gram-positive), *E. coli* ATCC 25922, and *P. aeruginosa* ATCC 9721 (gram-negative).

#### Culture media

Trypticase soy agar (TSA) and trypticase soy broth were purchased from Difco®. Mueller–Hinton broth and Brain–heart infusion broth (BHI were purchased from Merck®.

#### Collection and storage of microorganisms

The freeze-dried strains were hydrated with approximately 0.5 mL of sterile saline (0.9% NaCl), transferred to test tubes containing sterilized TSA, and then inclined and incubated at 32.5°C ± 2.5°C for 24 h. After the incubation, the tubes containing the microorganisms were stored at 4.0°C ± 0.5°C.

#### Inocula standardization

From the stock cultures stored at 4°C, the bacteria were subcultured in grooves on the surface of the TSA, inclined and incubated at 32.5°C ± 2.5°C for 24 h. A microorganism suspension was prepared using sterile saline (0.9% NaCl), adjusting the inoculum concentration to 1.0 × 10^8^ CFU/mL. The readings were performed using Densimat®, a photometer used to determine the optical density (OD) of bacterial inocula, providing results equivalent to the McFarland standard scale.

#### Determination of the minimum inhibitory concentration

The *C. brasiliense* supercritical CO_2_ extract (CBSE) and 20% ethanol solution were evaluated. The minimum inhibitory concentration (MIC) was determined by the classical method of successive dilution, adapted to 96-well microplates. The whole procedure was performed in triplicate and the results were considered valid when the positive control (inoculated culture medium) showed microbial growth and the negative control (culture medium and Ciprofloxacin 0.1% w/v) showed no growth [25-27].

### Antiseptic activity

Based on test results of MIC, CBSE was added to the formulations used in the antiseptic activity evaluation at a concentration of 2.5%.

#### Formulation preparation

Liquid soap and hand lotion used in the antiseptic activity evaluation were prepared in laboratory scale. For the liquid soap, the following raw materials were used: Sodium laureth-2 sulfate (25.0%), Cocamide DEA (5.0%), Cocamidopropyl betaine (2.0%), PEG-120 methyl glucose dioleate (2.0%), Phenoxyethanol (0.3%), Methylparaben (0.01%), Butylparaben (0.002%), Ethylparaben (0.002%), Propylparaben (0.018%), Water q.s. 100%, and Citric Acid q.s. pH 6.0. The hand lotion was prepared from the following materials: Cetearyl alcohol (5.0% w/w), Mineral oil (4.0% w/w), Glycerin (2.0% w/w), *Astrocarym murumuru* seed butter (2.0%), Lanolin alcohol (1.0% w/w), Phenoxyethanol (0.38%), Methylparaben (0.003%), Butylparaben (0.002%), Ethylparaben (0.002%), Propylparaben (0.018%), and Water q.s. 100%. The CBSE was added at a final concentration of 2.5% to both formulations. The liquid soap and hand lotion were also prepared without the extract, for use as a no-extract control.

#### Microbiological quality control of the formulations

Microbiological quality of the formulations was assessed according to the methods recommended by Pinto *et al*. [28] in order to avoid further contamination of the formulations.

#### Evaluation of antiseptic activity

Antiseptic activity was assessed by the plate inhibition method. Inhibition assays were performed against *S. aureus* ATCC 10390 (gram-positive), *E. coli* ATCC 25922 and *P. aeruginosa* ATCC 9721 (gram-negative). The previously standardized inoculum of each microorganism (0.2 mL) was added to the culture medium and distributed in Petri dishes. After solidification of the culture medium, the central region of the plate containing the culture medium was removed using sterile metallic cylinders with 1.5 cm diameter and filled in with 100 μL of the formulations containing the extract. The plates were incubated at 32.5°C ± 2.5°C for 48 h.

The same procedure was repeated using the formulations without the extract, and sterile saline (0.9% NaCl). All tests were performed in triplicate and the mean diameters of the inhibition zones were recorded.

### Antioxidant activity

#### Human fibroblast culture

Human fibroblasts (Human adult dermal fibroblasts, CC-2511, Clonetics, Cambrex/Lonza, USA) were seeded in 75 cm^2^ bottles (Corning Inc., USA) and grown in a wet incubator at 37°C in the presence of 5% CO_2_, using RPMI 1640 culture medium with 10% Fetal Bovine Serum. When 80%–90% confluence was reached, 50,000 cells/well were seeded in 24-well microplates (Nunc, USA) for further incubation with the samples, and evaluation of the proposed mediator.

#### Incubation with the extract

Cell cultures were incubated with four concentrations of CBSE (0.2, 0.1, 0.05, and 0.025% (w/v)) previously determined to be noncytotoxic using the XTT technique. The cell cultures remained in contact with 10 μL of the extract at these concentrations for 48 h, followed by the collection of the cell lysate.

#### Evaluation of antioxidant activity

The antioxidant concentration in the cell lysate was measured using a commercially available test kit (Sigma-Aldrich Inc., St. Louis, MI). This test is based on the formation of myoglobin ferryl radical from metmyoglobin and hydrogen peroxide. Myoglobin ferryl radical oxidizes 2,2′-azino-bis (3-ethylbenzthiazoline-6-sulfonic acid) (ABTS) to produce the radical cation ABTS^+^, which is a soluble chromogen that exhibits green coloration and can be determined in a spectrophotometer at 405 nm. Trolox (vitamin E-6-hydroxy-2,5,7,8-tetramethylchroman-2-carboxylic acid; Sigma-Aldrich Inc., St. Louis, MI) was used as a standard for measuring the antioxidant concentration. Results are represented in OD units.

The test kit comprised a buffer solution, a myoglobin solution, and an ABTS substrate solution. The ABTS substrate working solution was prepared by adding 25 μL from a 3% hydrogen peroxide solution to a 10 mL ABTS substrate solution.

The assay was performed in 96-well microplates. Wells containing cell lysate were treated with 20 μL of the myoglobin solution, followed by 150 μL of the ABTS substrate working solution. The microplates were incubated for 5 min at room temperature, following which the absorbance of each well was determined at 405 nm using a microplate reader (Multiscan MS, Labsystems, Helsinki, Finland).

Following the same procedure as that adopted for the test sample, 10 μL of the Trolox standard was used as a positive control.

#### Statistical analysis

One-way analysis of variance, followed by Dunnett’s test, was used to compare data among all groups. *P* values <0.05 were considered statistically significant.

## Results and discussion

### Screening of the crude extract

The terpenoid fraction was identified with anisaldehyde reagent, which produced spots having different shades of pink and violet. These spots were also observed with standard H_2_SO_4_, antimony chloride reagent, and 0.2% KMNO_4_.

The phenolic fraction was identified after acid hydrolysis of the dried ground plant extract. Acid hydrolysis was performed with 2 M HCl for 0.5 h. The product workup created an organic fraction that was analyzed by thin layer chromatography. After separation on silica gel using 45% ethyl acetate in hexane, blue spots were detected with Folin reagent.

### Antimicrobial activity

The present study was conducted to investigate the antimicrobial activity of CBSE for use as an active ingredient in antiseptic formulations. The extract was chosen for its good activity [15] and with the aim of providing cheaper formulations.

The MIC of CBSE was tested against two gram-negative and one gram-positive bacterial species (Table 1). The crude extract was found active against all bacteria tested. *S. aureus* is a pathogen which is known to cause infectious disorders of the skin [29,30]. Thus, CBSE may be used as agent for the treatment of skin disorders.

**Table 1:** Values of Minimum inhibitory concentration (MIC) of CBSE and Ethanol solution

**Microrganism**	**Samples**			
	**CBSE (%w/v)**	**CBSE (mg/mL)**	**ES (%w/v)**	**ES (mg/mL)**
*Escherichia coli* (ATCC 25922)	1.25	11.25	2.50	25.00
*Pseudomonas aeruginosa* (ATCC 9027)	2.50	22.50	5.00	50.00
*Staphylococcus aureus* (ATCC 6538)	1.25	11.25	10.00	100.00

ES: Ethanol solution (20%).

### Values of Minimum Inhibitory Concentration (MIC) of CBSE and Ethanol solution

The MIC is defined as the lowest concentration of a formulation that visibly inhibits *in vitro* microorganism growth*.* The summarized values indicate the concentration of CBSE that inhibited the growth of all microorganisms in the dilution tests. These results show that the inhibition values for *E. coli and S. aureus* growth are equivalent and are lower than those reported for *P. aeruginosa.*

The results confirm that CBSE exhibits antimicrobial activity, as previously demonstrated by Paula-Junior *et al*. [15]. They found that a hydroethanolic extract of *C. brasiliense* leaves exhibited MIC values of 4 mg/mL only for *E. coli* and *S. aureus.* Because the medium became cloudy with increasing extract concentration, they could not verify a MIC for *P. aeruginosa*. In our study, we found that a concentration of 11.25 mg/mL inhibits the growth of *E. coli* and *S. aureus* and a concentration of 22.50 mg/mL inhibits the growth of *P. aeruginosa*.

We also determined a MIC of 20% for ethanol, which indicates that there was no interference from the solvent in the results presented. For all microorganisms tested, the MIC values for CBSE are lower than the MIC values for the ethanol concentrations tested (Table 1).

The results of antimicrobial activity were validated when compared to the controls. In the positive control (no extract) there was no verified reduction of microbial growth, proving the viability of the strains, and no growth was noted in the negative controls, demonstrating the susceptibility of the microorganisms to the control antibiotic.

The liquid medium dilution technique is used more frequently than the agar diffusion method when evaluating the antimicrobial activity. This technique has been the primary method used to determine the MIC of antibiotics, antiseptics, and preservatives [31].

### Antiseptic activity

Formulations were prepared with CBSE as an antiseptic agent, not as a preservative. The microbiological quality control of the formulations showed that the total viable count of microorganisms was of < 10 CFU/mL^−1^. This means that the formulation could be approved for use in baby and eye products, because it did not exceed the official limit of < 5 × 10^2^ CFU/g^−1^ or mL^−1^ for these products, and of < 10^3^ CFU/g^−1^ or mL^−1^ for any other cosmetic product [24].

### Antiseptic activity of CBSE

Both formulations containing CBSE proved to be effective antiseptics against all three microorganisms tested (Table 2). A product is classified as antiseptic when it produces an inhibition zone of 8 mm [31,32].

**Table 2:** Antiseptic activity of CBSE

**Microorganism**	**Inhibition zone (mm)**				
	**LSCE**	**HLCE**	**LSWE**	**HLWE**	**C (-)**
*Escherichia coli* (ATCC 25922)	13.0	12.6	2.9	1.9	-
*Pseudomonas aeruginosa* (ATCC 9027)	9.0	8.2	2.1	2.0	-
*Staphylococcus aureus* (ATCC 6538)	11.0	11.0	2.3	1.9	-

LSCE: Liquid soap containing extract.

HLCE: Hand lotion containing extract.

LSWE: Liquid soap without extract.

HLWE: Hand lotion without extract.

C (-): Negative control (sterile saline 0.9%).

Previous studies demonstrated that the main constituents of *C. brasiliense* are flavonoids and terpenoids, chemical compounds identified as antimicrobial agents [15,33-36].

Flavonoids can interact with the cytoplasmic membrane, inhibiting its function and jeopardizing cellular integrity; they can also inhibit the synthesis of nucleic acids and interrupt bacterial metabolism [37]. Terpenoids are Generally Recognized as Safe and have been found to inhibit the growth of cancerous cells, decrease tumor size, decrease serum cholesterol levels, and decrease microorganism concentrations [38,39]. Terpenoids have lipophilic characteristics that affect the cytoplasmic membrane stability, leading to the loss of cellular enzymes and nutrients [40].

The antimicrobial properties of plant extracts have generated interest in determining the structure of phytochemicals and examining their activity as broad-spectrum antimicrobial agents. The antibacterial activity of *C. brasiliense* could be attributed to its chemical properties because the control samples did not present significant microbial growth inhibition (*P* < 0.05) compared with the test samples.

### Antioxidant activity

**Figure 1:**
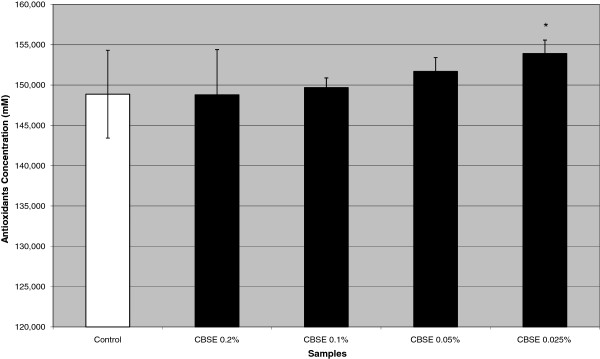
**Antioxidant concentration of Caryocar *brasiliense *supercritical extract (CBSE).** Antioxidant concentration (mM–relative to Trolox standard) in cell lysate treated with *Caryocar brasiliense* supercritical extract (0.2, 0.1, 0.05 and 0.025% w/v). The data represent the mean ± SD of 3 independent experiments. *P <0.05, compared to control (ANOVA, Dunnett).

Figure 1 shows the considerable antioxidant potential of CBSE. When applied to cell cultures at concentrations of 0.025% (w/v), it caused a statistically significant (*P* < 0.05) increase of approximately 3% in the antioxidant concentration in cell lysates, when compared with the control group. Chemical constituents like flavonoids and terpenoids present in *C. brasiliense* contribute to this activity [41-44]. These experiments demonstrate the extract’s antioxidant capacity, suggesting its use in cosmetics with anti-aging action.

The search for substances that can either slow or reverse the aging process is a constant subject of research and development in the cosmetics and dermatological fields. Free radicals, such as superoxide and hydroxyl radical, are formed by the oxidative phosphorylation process and cause damage to lipid components, proteins, and nucleic acids. To reverse the detrimental oxidative process, cells use an endogenous defense mechanism called redox homeostasis, which consists of a balance between production and elimination of free radicals by various antioxidants and enzymes.

### Antioxidant concentration of *C. brasiliense* supercritical extract (CBSE)

The antioxidant activity of a pure substance, or a mixture of compounds such as extracts, cannot be measured directly because this activity involves diverse mechanisms, especially those related to the prevention of the chain initiator, binding to transition metals, and peroxide decomposition [45]. Instead, their antioxidant activity is measured through their effects on a substrate or system capable of being monitored.

Furthermore, most assessment methods for antioxidants use oxidation processes involving the addition of an initiator (such as temperature, agitation, or a partial O_2_ pressure), a process accelerator (e.g., a transition metal or exposure to light), and a specific source of free radicals. These radicals are then oxidized under standard conditions, and the degree of oxidation, or its extension, is measured [46].

The method used to determine the antioxidant potential of CBSE involved ABTS radical production from its precursor, ABTS. ABTS radical is a very chemically stable chromophoric compound with high water solubility, a maximum absorbance at 414 nm, and secondary absorbances at 645, 734, and 815 nm [47].

The ABTS radical method exhibits excellent stability and is one of the fastest methods for the evaluation of antioxidant activity. It provides reproducible results, maximum absorption, and good solubility. This method is used for hydrosoluble and liposoluble samples, which gives it an advantage over other methods. It has been used in food products, pure compounds, and plant extracts [48-50].

The ABTS/myoglobin/H_2_O_2_ system, also known as the Total Activity Method or the Rice–Evans method, is the best known method for the measurement of antioxidant activity. It was the first method to use ABTS as an oxidation substrate. It formed the basis for the emergence of other methods using the same radical [47].

## Conclusions

Under the tests conditions used, we concluded that the extract of *C. brasiliense* (Pequi) obtained by supercritical CO_2_ extraction has antimicrobial and antioxidant activities. This suggests that the extract may be useful in the development of personal care products, primarily for antiseptic skin products that inactivate, reduce, prevent, or arrest the growth of microorganisms with the inherent intent to mitigate or prevent disease as well as products that minimize damage caused by free radicals.

## Competing interests

The authors declare that they have no competing interests. There is no financial relationship with other people or organizations in implementation, analysis or financing of this study.

## Authors’ contributions

MAF and PM carried out the phytochemical study. LFBA and PGM carried out the antibacterial and antioxidant experiments, wrote the paper and evaluated the data. All authors read, reviewed and approved the final manuscript.

## Pre-publication history

The pre-publication history for this paper can be accessed here:

http://www.biomedcentral.com/1472-6882/14/73/prepub
