# Correction: Caryocar brasiliense supercritical CO_2_ extract possesses antimicrobial and antioxidant properties useful for personal care products

**DOI:** 10.1186/1472-6882-14-154

**Published:** 2014-05-21

**Authors:** Lilian FB Amaral, Patricia Moriel, Mary Ann Foglio, Priscila G Mazzola

**Affiliations:** 1 Department of Clinical Pathology, Faculty of Medical Sciences, University of Campinas (FCM-UNICAMP)-Rua Tessália Vieira de Camargo, 126 Campinas, SP, Brazil; 2 CPQBA-Multidisciplinary Center of Chemical, Biological and Agricultural Researches, University of Campinas (UNICAMP)-Rua Alexandre Cazellato, 999, Vila Betel, Paulínia, SP, Brazil; 3 Rua Horácio Cenci, 345, Apto 32, Campolim, 18047-800 Sorocaba, São Paulo, Brazil

## Correction

After publication of the original manuscript [1], additional funding was secured which the authors wish to acknowledge. The amended Acknowledgements section for this paper therefore reads:

## Pre-publication history

The pre-publication history for this paper can be accessed here:

http://www.biomedcentral.com/1472-6882/14/154/prepub
